# Impact of the Play Active policy intervention on early childhood educator's sedentary behaviour‐related practices, psychosocial influences and meeting policy recommendations: Results from a pragmatic cluster randomized trial

**DOI:** 10.1111/ijpo.70005

**Published:** 2025-03-11

**Authors:** Hayley Christian, Andrea Nathan, Emma Adams, Stewart G. Trost, Jasper Schipperijn

**Affiliations:** ^1^ The Kids Research Institute Australia The University of Western Australia Perth Western Australia Australia; ^2^ School of Population and Global Health The University of Western Australia Perth Western Australia Australia; ^3^ School of Human Movement and Nutrition Sciences University of Queensland Brisbane Queensland Australia; ^4^ Department of Sports Science and Clinical Biomechanics University of Southern Denmark Odense Denmark

**Keywords:** childcare, implementation, intervention, physical activity, policy, sedentary behaviour

## Abstract

**Background:**

High levels of sedentary behaviour are associated with poor child health outcomes such as obesity. Early childhood education and care (ECEC) services are a key intervention setting. Most ECEC policy‐based interventions focus on children's nutrition and physical activity with few aimed at children's sedentary behaviour.

**Objective:**

To evaluate the effect of the Play Active ECEC policy intervention on educator adherence to sedentary behaviour policy recommendations, educator's practices and educator psychosocial influences related to children's sedentary behaviour.

**Methods:**

Pragmatic cluster randomized trial in 81 ECEC services in Perth, Western Australia. Services implemented the Play Active policy over three months. Outcomes were educator‐reported changes in adherence to sedentary behaviour policy recommendations, practices and psychosocial influences related to children's sedentary behaviour. Analysis involved descriptive statistics and generalized linear mixed‐effects models.

**Results:**

Adherence to sedentary behaviour policy recommendations and educator's practices and psychosocial influences related to children's sedentary behaviour was high at baseline and did not significantly change in response to the Play Active policy intervention.

**Conclusions:**

Educators appeared to adhere to best‐practice guidelines for children's sedentary behaviour in ECEC. Clear evidence informed policy, standards and legislation to maintain children's low levels of sedentary behaviours in ECEC is warranted.

**Abbreviations**
BC: British Columbia
CONSORT: Consolidated Standards of Reporting Trials
ECEC: early childhood education and care
EPAO: Environment and Policy Assessment and Observation
GLMM: generalized linear mixed effects models
NAPSACC: Nutrition and Physical Activity Self‐Assessment for Child Care
NHMRC: National Health and Medical Research Council
OR: odds ratio
RTP: Research Training Program
WA: Western Australia

## INTRODUCTION

Over 40 million children under the age of five are overweight or obese, with rates of obesity increasing.^1^ Overweight and obesity in childhood have serious short‐ and long‐term consequences on a child's physical and mental health.^2^ High levels of sedentary behaviour such as recreational screen time, is associated with poorer child health outcomes including lower fitness, poorer cardiometabolic health, shorter sleep duration, unfavourable measures of adiposity and poorer mental health.^3^, ^4^, ^5^, ^6^ It is vital that healthy behaviours such as regular daily physical activity and minimizing sedentary time are established early in life.^2^ The World Health Organization's 24‐h movement guidelines recommend young children (3–5 years) accumulate: (1) at least 180 min of total physical activity per day, including at least 60 min of energetic (moderate to vigorous) play, (2) no more than 60 min of sedentary screen time per day, and (3) 10–13 h of sleep per day.^7^ Yet, young children spend a substantial proportion of their time sedentary, with many exposed to screens well before the age of two and for more than the recommended maximum of 60 min per day for 3–5 year olds.^8^, ^9^, ^10^

Early childhood education and care (ECEC) services are a key setting to reach children in their early years, due to the large numbers of children attending these services for regular and prolonged periods of time.^11^ A systematic review and meta‐analysis showed that while young children attending ECEC have the potential to be highly active during outdoor play sessions, many are reported to engage in substantial amounts of sedentary time (approximately 55% of attendance time).^12^ Moreover, a recent systematic review of 29 studies of children's device‐measured sedentary time at ECEC showed on average children spent 30–46 min per hour at ECEC sedentary.^13^ Yet, other device‐based studies show children's sedentary bouts in ECEC last less than 5 min at a time.^14^, ^15^ Overall, compared to physical activity, there is substantially less research on children's sedentary behaviour in the ECEC setting.

A significant barrier to reducing the amount of time children are sedentary in ECEC is the lack of evidence‐informed guidance on the maximum amount of sedentary (including screen) time children should be permitted while attending ECEC.^16^ A recent international scoping review identified only 51% of ECEC policy documents included sedentary time recommendations, and there was substantial variation in the recommended maximum amount of sedentary time, ranging from 15 to 60 min per day at ECEC.^17^ Reassuringly, no policies permitted screen time for children under 2 years; however, between 20 and 120 min of screen time per day was permitted for children 2 years or older.^17^

ECEC‐based policy interventions have predominantly focused on children's nutrition and physical activity behaviours with fewer specifically aimed at children's sedentary behaviour.^18^ Furthermore, few interventions have focused on ECEC‐specific recommendations on the maximum amount of sedentary time children should have while attending ECEC. Of the handful of small intervention studies to evaluate the implementation of ECEC‐specific sedentary recommendations, four involved evaluations of changes to local childcare quality standards or licensing to incorporate guidance on children's nutrition and/or physical activity (and sedentary) behaviour in ECEC.^19^, ^20^, ^21^, ^22^, ^23^ Three intervention studies had relatively small sample sizes, with less than a total of 10 participating services^19^, ^21^, ^24^ and one had no sedentary outcome.^25^

Of the two intervention studies using device‐based measures of children's sedentary time, neither reported a significant change post‐intervention.^19^, ^20^, ^24^ For example, the Childcare PLAY policy was implemented over an 8‐week period and evaluated using a cluster randomized controlled trial in nine (five intervention) childcare centres in London, Ontario.^24^ The PLAY policy included eight statements targeting physical activity/outdoor play (six statements) and sedentary/screen time (two statements). The lack of significant findings may in part be due to device‐based measurement protocols only being able to capture movement behaviour and not actual screen use. This study was limited by its small sample, lack of policy key features (e.g., rationale, links to relevant legislation, procedures for implementation support) and absence of specific recommendations on the maximum amount of daily sedentary/screen time children should have at ECEC and implementation supports such as supporting resources, training and policy monitoring and review.^18^, ^24^, ^26^ Further research is needed to determine the impact of sedentary‐based ECEC policy interventions on the factors influencing children's sedentary behaviour.

Five ECEC sedentary policy intervention studies to date have measured change in educator sedentary practices, with four using the Environment and Policy Assessment and Observation (EPAO)^21^, ^22^, ^27^, ^28^ and another using intervention‐specific measures of educator sedentary practices.^23^ For example, using a repeat cross‐sectional design, centre‐level change in seven sedentary‐based policies and practices was examined approximately 12 months after the implementation of mandatory Active Play Standards in British Columbia (BC), Canada.^23^ Post‐implementation of the new Active Play Standards, there was a significant increase in centres with policies related to the amount of screen time, breaking up prolonged sitting and educator modelling of screen time, as well as a significant increase in educator practices related to spending 30 min or less on screens daily.^23^ However, as this natural policy experiment did not include a comparison group, it precludes the ability to infer causal relationships between the introduction of the Active Play Standards and improvements in centre sedentary behaviour‐related policies and practices.

Overall, of the few sedentary‐focused policy interventions in ECEC, all suffer from methodological limitations. Moreover, given behaviour change pathways and the multiple levels of influence on a child's sedentary behaviour at ECEC (e.g., policy and physical environment; educator intrapersonal and interpersonal factors and practices; child‐level factors), educator psychosocial factors should be included as outcomes in ECEC policy‐based interventions. Psychosocial factors encompass the social, cultural and environmental factors influencing an individual's health and the interaction between individual‐level factors such as self‐efficacy and social factors such as social support.^29^, ^30^ There is some evidence that educator physical activity‐related self‐efficacy may be important for supporting movement opportunities for young children while attending ECEC^31^, ^32^, ^33^, ^34^; however, further research is required to confirm these findings for children's sedentary behaviour in ECEC. Additionally, to assess the shorter‐term impact of policy interventions, changes in educator practices and psychosocial factors as they relate to children's sedentary behaviour are needed, in addition to device‐based measures of child sedentary time. The Play Active ECEC‐specific policy intervention applied this approach. Play Active is a physical activity and sedentary behaviour policy intervention to improve ECEC educator's physical activity and sedentary practices. The central component of Play Active is an evidence‐informed policy template which includes 25 practices to support nine age‐specific recommendations on the amount of physical activity and sedentary time, including screen time, young children should do while in ECEC.^16^, ^35^ The aim of this paper was to evaluate the effect of the Play Active intervention on educator adherence to sedentary behaviour policy recommendations, educator's practices and educator psychosocial influences related to young children's sedentary behaviour.

## METHODS

The trial protocol has previously been described in full.^35^ The physical activity‐related methods and findings from the study have previously been published.^26^ This paper follows the Consolidated Standards of Reporting Trials (CONSORT) checklist,^36^ including cluster^37^ and pragmatic extensions.^38^ Ethics approval was obtained from The University of Western Australia Human Research Ethics Committee (RA/4/20/6120 approved 19 May 2020). The trial was registered through the Australian New Zealand Clinical Trials Registry (reference number 12620001206910, registered 13 November 2020).

### Trial design and setting

Play Active was evaluated using a pragmatic cluster randomized trial design.^35^ The trial was conducted between January 2021 and March 2022 and included 81 long‐day ECEC services in Perth, Western Australia. At recruitment, there were a total of 557 long‐day ECEC services in the study area.

### Participants and recruitment

ECEC services were recruited via an ‘Expression of Interest’ available on a study partner's website. Ineligibility criteria included: services catering exclusively to children requiring specialist care, mobile preschools, Department of Education and Communities preschools, services already involved in another trial, and services with or expecting a significant management change in the last/next three months. Eligible service directors and educators were provided with study information and provided consent to participate.^35^

### Randomization and blinding

At the conclusion of baseline data collection, 81 ECEC services were randomly allocated to either the intervention (*n* = 41) or wait‐listed comparison (*n* = 40) groups using a central randomization procedure (in Microsoft Excel). To avoid contamination between services, groups of services within the same provider in close geographical proximity to each other were randomly allocated to the same group. The research team member randomizing services was not involved in recruitment, data collection or intervention delivery.

### The Play Active intervention and implementation support strategies

Play Active has been described in detail elsewhere.^16^, ^35^ Briefly, the central component of Play Active was an evidence‐informed physical activity and sedentary behaviour policy template^35^ which was emailed to intervention ECEC service directors after they completed baseline data collection (April–June 2021). The policy template included two overarching key statements and nine age‐specific recommendations on the amount of physical activity and sedentary time (including screen time), for young children while in ECEC. To support achieving these recommendations, the policy template also included 25 physical activity and sedentary‐related practices specific to management and educators (*n* = 14), the physical environment (*n* = 4), parent and carer engagement (*n* = 5), and policy monitoring and review (*n* = 2).^16^, ^26^, ^35^ One of the two key statements (‘Limit sedentary behaviours in young children’), five of the nine age‐specific recommendations (e.g., ‘Sedentary screen time for purposes other than learning will not be allowed’) and three of the 25 practices (e.g., ‘Break up prolonged periods of sedentary behaviour’) related to children's sedentary behaviour.

To facilitate policy implementation in ECEC services, six implementation support strategies were provided: (i) policy personalisation; (ii) policy review and approval; (iii) resource guide (practical tips and activity suggestions with resources mapped to the 25 practices within the policy); (iv) brief energetic play assessment tool (for monitoring young children's physical activity levels, provided within the resource guide, based on an instrument developed for family day care)^39^; (v) educator professional development and training (training on fundamental movement skills and active play‐based learning provided by Play Active partners); and (vi) project officer implementation support (weekly follow‐up (phone/email) to complete the policy review and mid‐implementation prompt (phone) to confirm policy implementation had commenced).^35^ Directors were given up to 5 months to complete policy personalisation and implementation. This included up to 2 months to personalize the physical activity and sedentary behaviour policy template. Directors were asked to return their policy via email to the research team for review and approval, conducted independently by two project officers. Once approved, services were asked to start implementing their policy and were provided with the remaining four implementation support strategies. Wait‐listed comparison services were asked to continue their usual physical activity and sedentary behaviour practices.

### Data collection

Baseline data collection occurred from January to June 2021 using educator‐completed online and paper surveys. Post‐intervention data collection was conducted from September 2021 to March 2022. Survey items aligned with the Play Active policy recommendations and practices.

#### Adherence to policy recommendations, educator practices and educator psychosocial influences related to children's sedentary behaviour

To determine if the sedentary time policy recommendations were being met, educators reported the longest time children were expected to remain seated at any one time (seven‐point scale, <10; 10–19; 20–29; 30–39; 40–49; 50–59; 60+ min at any one time). Educators also reported the amount of screen time they allowed children each day (six‐point scale, 0; 1–14; 15–29; 30–44; 45–59; 60+ min per day).

Three educator practices relating to children's sedentary behaviour were reported: (1) *I avoid sitting while supervising outside play*; (2) *I increase screen time use as a reward for good behaviour or take it away for bad behaviour*; (3) *When screen time is offered, children are given the opportunity to do an alternative activity* (all six‐point scales, never; rarely; sometimes; often; very often; always). Items were based on established, validated instruments (Nutrition and Physical Activity Self‐Assessment for Child Care [NAPSACC])^40^ and Environment and Policy Assessment and Observation (EPAO)—Self Report,^41^ and had acceptable test–retest reliability in the Australian ECEC context.^42^

In addition, six items measuring educator's self‐efficacy, motivation and support regarding children's sedentary behaviour were measured (seven‐point scale, strongly disagree to strongly agree). Items were context‐specific to the Play Active program and consistent with standard items used to measure psychosocial factors.^43^, ^44^, ^45^ The self‐efficacy and motivation items have been shown to have good acceptability, internal consistency and test–retest reliability.^43^ The social support items have been shown to have good reliability and evidence of criterion‐related and construct validity.^45^

#### Educator characteristics

Educator level of education was dichotomised into high school, trade or diploma, and university education. Educators also reported their year of birth, from which age was derived. Educators were asked if they were female, male or self‐described. Additionally, educators reported at baseline the frequency they had received professional development on screen and sitting time recommendations for young children (never, less than once/year, once/year; >once/year). These two professional development items were combined into one item by taking the higher frequency.

### Analysis

The effect of the intervention on educator adherence to sedentary behaviour policy recommendations, educator sedentary behaviour‐related practices, and educator psychosocial influences was analysed using generalized linear mixed effects models (GLMM) that included fixed effects for group (intervention vs. wait‐listed comparison), time (post‐intervention vs. baseline) and time‐by‐group interaction; random intercept effects for individual educators nested within services; and an exchangeable correlation structure. Fixed effects were also included for educator age and highest level of education. All educators were included in the analysis if they provided outcome data for at least one of the measurement points. Data preparation and cleaning were undertaken in SAS 9.4 and GLMMs in Stata 17 using *meglm* with a binomial distribution and logit link for dichotomous outcomes and ordinal distribution and logit link for ordinal outcomes.

## RESULTS

### Educator characteristics

**FIGURE 1:**
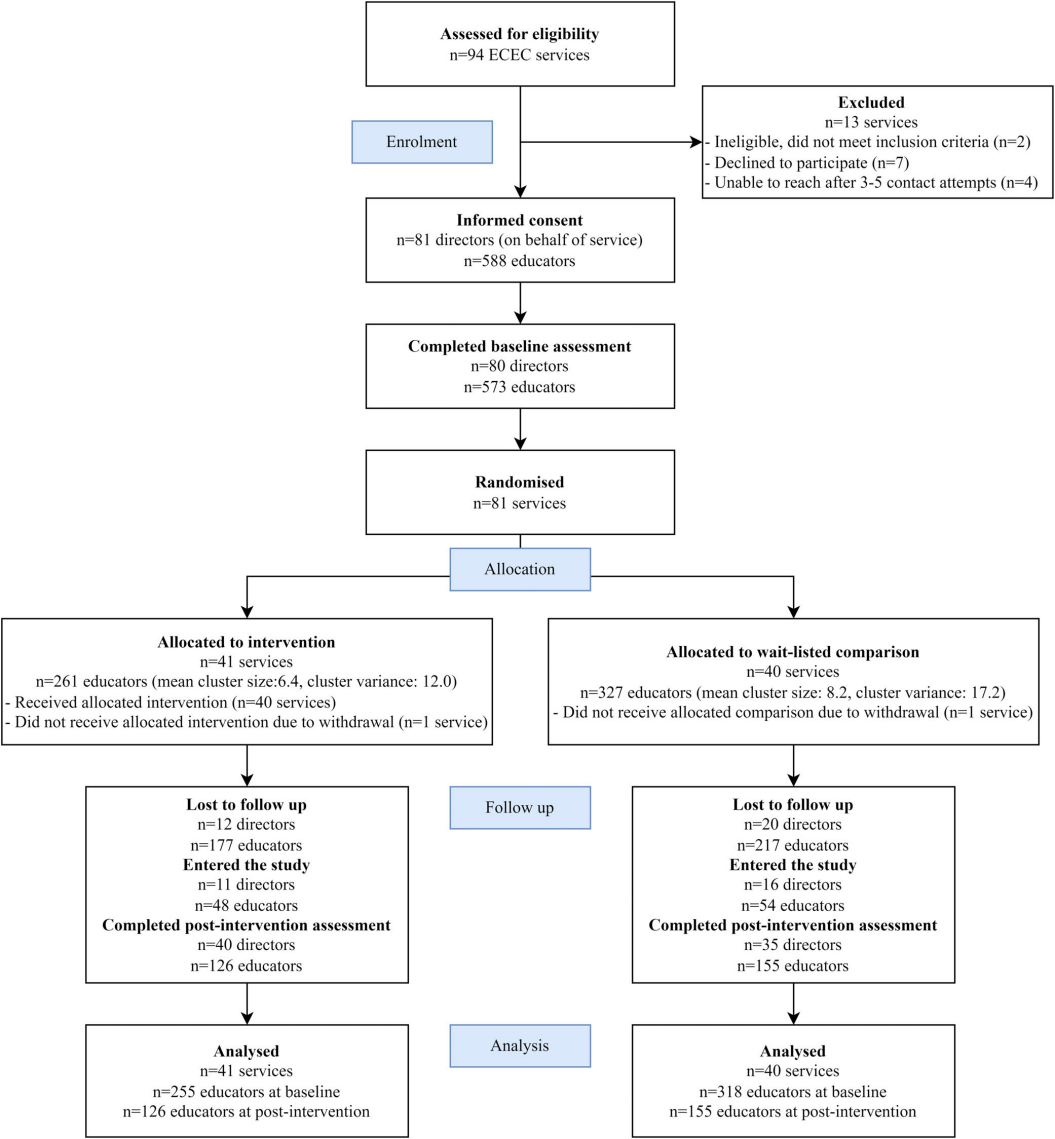
Play Active participant flow chart. ECEC, early childhood education and care.

The flowchart of participants in the pragmatic trial is presented in Figure 1. In total, 573 and 271 educators completed the baseline and post‐intervention surveys, respectively.

As reported in Table 1, almost all educators in the intervention and wait‐listed comparison groups were female, and about one‐quarter of educators had university qualifications (28.3% in wait‐listed comparison group; 23.9% in intervention group). There were no overall differences in educator characteristics between the wait‐listed comparison and intervention groups.

**TABLE 1:** Educator characteristics by experimental group.

	Wait‐listed comparison group	Intervention group
Age	*n* = 363	*n* = 300
Years, median (IQR)	34.0 (15.0)	33.0 (15.0)
Gender	*n* = 368	*n* = 300
Female, *n* (%)	364 (98.9)	296 (98.7)
Qualification level	*n* = 367	*n* = 301
School, *n* (%)	85 (23.2)	72 (23.9)
Diploma, *n* (%)	178 (48.5)	157 (52.2)
University, *n* (%)	104 (28.3)	72 (23.9)
Received professional development training on limiting children's sedentary behaviours^a^	*n* = 284	*n* = 234
Never, *n* (%)	98 (34.5)	96 (41.0)
Less than 1 time per year, *n* (%)	84 (29.6)	53 (22.6)
1 time per year, *n* (%)	61 (21.5)	45 (19.2)
More than 1 time per year, *n* (%)	41 (14.4)	40 (17.1)

*Note*: Differences between groups were tested for statistical significance using chi‐square and Wilcoxon tests. No total differences between wait‐listed comparison and intervention groups were statistically significant.

Abbreviation: IQR, interquartile range.

^a^ Received professional development in the last 2 years on limiting long periods of sitting for young children or recommended amounts of screen time for young children.

### Educator practices and psychosocial influences related to children's sedentary behaviour

Descriptive statistics for all educator outcomes by experimental group are presented in Table 2. Additionally, Table 2 presents the adjusted model coefficients; a significant group‐by‐time interaction indicates that the change from baseline to post‐intervention for intervention educators was statistically different from the change in wait‐listed comparison educators. Results are reported as coefficients and odds ratios (OR); the OR can be interpreted as the effect of the independent variable on the odds of being in a higher category of the dependent variable.

**TABLE 2:** Sedentary behaviour outcome descriptives by group and time, and group by time estimated coefficients (B) and odds ratios (OR).

	Wait‐listed comparison		Intervention		Group*time B (95% CI)	OR (95% CI)	ICC
	Baseline, *n* (%)	Post‐intervention, *n* (%)	Baseline, *n* (%)	Post‐intervention, *n* (%)			
Policy recommendations							
Meets policy recommendation of not being seated for more than 60 min at a time	*n* = 296	*n* = 145	*n* = 241	*n* = 118	No estimates		
Yes	296 (100.0)	144 (99.3)	236 (97.9)	114 (96.6)			
Outside of sleep and meal times, the longest children in my care are expected to remain seated at any one time is	*n* = 296	*n* = 145	*n* = 241	*n* = 118	0.2 (−0.5, 0.8)	1.2 (0.6, 2.3)	0.03
<10 min	148 (50.0)	62 (42.8)	121 (50.2)	45 (38.1)			
10–19 min	104 (35.1)	56 (38.6)	68 (28.2)	41 (34.7)			
20–29 min	28 (9.5)	15 (10.3)	30 (12.4)	21 (17.8)			
30–39 min	12 (4.1)	10 (6.9)	12 (5.0)	4 (3.4)			
40–49 min	2 (0.7)	0 (0.0)	4 (1.7)	2 (1.7)			
50–59 min	2 (0.7)	1 (0.7)	1 (0.4)	1 (0.8)			
60+ min	0 (0.0)	1 (0.7)	5 (2.1)	4 (3.4)			
The amount of screen time I provide each day to children is	*n* = 299	*n* = 140	*n* = 244	*n* = 117	0.3 (−0.7, 1.2)	1.3 (0.5, 3.2)	0.29
0 min	179 (59.9)	82 (58.6)	178 (73.0)	80 (68.4)			
1–14 min	101 (33.8)	51 (36.4)	61 (25.0)	31 (26.5)			
15+ min	19 (6.3)	7 (5.0)	5 (2.0)	6 (5.2)			
Educator practices							
I avoid sitting while supervising outside play	*n* = 294	*n* = 141	*n* = 244	*n* = 119	−0.3 (−0.9, 0.3)	0.7 (0.4, 1.4)	0.09
Mean (95% CI)	4.0 (3.8–4.2)	4.1 (3.8–4.4)	4.1 (3.9–4.3)	4.0 (3.7–4.3)			
Never	13 (4.4)	9 (9.4)	14 (5.7)	8 (6.7)			
Rarely	26 (8.8)	11 (7.8)	18 (7.4)	10 (8.4)			
Sometimes	90 (30.6)	32 (22.7)	54 (22.1)	31 (26.1)			
Often	44 (15.0)	25 (17.7)	46 (18.9)	18 (15.1)			
Very often	70 (23.8)	34 (24.1)	61 (25.0)	27 (22.7)			
Always	51 (17.3)	30 (21.3)	51 (20.9)	25 (21.0)			
I increase screen time as a reward for good behaviour or take it away for bad behaviour	*n* = 296	*n* = 141	*n* = 244	*n* = 118	0.8 (−0.5, 2.1)	2.2 (0.6, 8.4)	0.27
Mean (95% CI)	1.2 (1.1–1.3)	1.1 (1.0–1.2)	1.2 (1.1–1.3)	1.2 (1.1–1.3)			
Never	246 (83.1)	123 (87.2)	220 (90.2)	106 (89.8)			
Rarely	30 (10.1)	16 (11.3)	15 (6.1)	4 (3.4)			
Sometimes	19 (6.4)	2 (1.4)	7 (2.9)	6 (5.1)			
Often	0 (0.0)	0 (0.0)	0 (0.0)	2 (1.7)			
Very often	1 (0.3)	0 (0.0)	1 (0.4)	0 (0.0)			
Always	0 (0.0)	0 (0.0)	1 (0.4)	0 (0.0)			
When screen time is offered, children are given the opportunity to do an alternative activity^a^	*n* = 119	*n* = 60	*n* = 63	*n* = 37	−0.1 (−1.3, 1.1)	0.9 (0.3, 3.1)	0.07
Mean (95% CI)	4.3 (4.0–4.5)	4.3 (3.8–4.8)	3.9 (3.5–4.3)	4.1 (3.6–4.6)			
Never	8 (6.7)	2 (3.3)	4 (6.3)	1 (2.7)			
Rarely	7 (5.9)	1 (1.7)	6 (9.5)	2 (5.4)			
Sometimes	14 (11.8)	6 (10.0)	10 (15.9)	4 (10.8)			
Often	20 (16.8)	5 (13.3)	13 (20.6)	7 (18.9)			
Very often	17 (14.3)	5 (8.3)	5 (7.9)	4 (10.8)			
Always	53 (44.5)	38 (63.3)	25 (36.7)	19 (51.4)			
Psychosocial influences							
I feel able to break up prolonged sitting and limit the total amount of time toddlers and kindergarten children spend sitting	*n* = 279	*n* = 139	*n* = 229	*n* = 108	−0.3 (−1.0, 0.4)	0.8 (0.4, 1.5)	0.03
Mean (95% CI)	6.0 (5.9–6.1)	6.2 (6.1–6.4)	6.0 (5.9–6.2)	6.1 (6.0–6.3)			
Strongly disagree	1 (0.4)	0 (0.0)	0 (0.0)	1 (0.9)			
Disagree	1 (0.4)	1 (0.7)	2 (0.9)	0 (0.0)			
Somewhat disagree	2 (0.7)	0 (0.0)	0 (0.0)	0 (0.0)			
Neither agree nor disagree	17 (6.1)	5 (3.6)	14 (6.1)	3 (2.8)			
Somewhat agree	31 (11.1)	4 (2.9)	26 (11.4)	12 (11.1)			
Agree	146 (52.3)	80 (57.6)	114 (49.8)	56 (51.9)			
Strongly agree	81 (29.0)	49 (35.3)	73 (31.9)	36 (33.3)			
I feel able to ensure children in my care don't have any sedentary screen time	*n* = 280	*n* = 137	*n* = 229	*n* = 110	−0.6 (−1.3, 0.2)	0.6 (0.3, 1.2)	0.10
Mean (95% CI)	6.3 (6.1–6.4)	6.3 (6.1–6.5)	6.2 (6.1–6.4)	6.2 (6.0–6.4)			
Strongly disagree	0 (0.0)	1 (0.7)	3 (1.3)	1 (0.9)			
Disagree	2 (0.7)	1 (0.7)	2 (0.9)	1 (0.9)			
Somewhat disagree	2 (0.7)	1 (0.7)	1 (0.4)	0 (0.0)			
Neither agree nor disagree	12 (4.3)	5 (3.6)	12 (5.2)	6 (5.5)			
Somewhat agree	28 (10.0)	5 (3.6)	15 (6.6)	12 (10.9)			
Agree	97 (34.6)	54 (39.4)	74 (32.3)	37 (33.6)			
Strongly agree	139 (49.6)	70 (51.1)	122 (53.3)	53 (48.2)			
I am motivated to break up prolonged sitting and limit the total amount of time toddlers and kindergarten children spend sitting	*n* = 277	*n* = 135	*n* = 226	*n* = 110	−0.0 (−0.8, 0.7)	1.0 (0.5, 2.0)	0.02
Mean (95% CI)	6.2 (6.1–6.3)	6.3 (6.2–6.5)	6.3 (6.2–6.4)	6.3 (6.2–6.4)			
Strongly disagree	0 (0.0)	0 (0.0)	0 (0.0)	0 (0.0)			
Disagree	0 (0.0)	1 (0.7)	0 (0.0)	0 (0.0)			
Somewhat disagree	2 (0.7)	0 (0.0)	1 (0.4)	0 (0.0)			
Neither agree nor disagree	8 (2.9)	4 (3.0)	5 (2.2)	3 (2.7)			
Somewhat agree	17 (6.1)	4 (3.0)	20 (8.8)	7 (6.4)			
Agree	143 (51.6)	68 (50.4)	103 (45.6)	54 (49.1)			
Strongly agree	107 (38.6)	58 (43.0)	97 (42.9)	46 (41.8)			
I am motivated to ensure children in my care don't have any sedentary screen time	*n* = 279	*n* = 140	*n* = 226	*n* = 111	−0.1 (−0.9, 0.7)	0.9 (0.4, 2.0)	0.04
Mean (95% CI)	6.3 (6.2–6.4)	6.3 (6.1–6.5)	6.4 (6.2–6.5)	6.2 (6.0–6.4)			
Strongly disagree	0 (0.0)	1 (0.7)	0 (0.0)	1 (0.9)			
Disagree	1 (0.4)	1 (0.7)	1 (0.4)	0 (0.0)			
Somewhat disagree	1 (0.4)	0 (0.0)	2 (0.9)	1 (0.9)			
Neither agree nor disagree	11 (3.9)	6 (4.3)	6 (2.7)	6 (5.4)			
Somewhat agree	17 (6.1)	9 (6.4)	16 (7.1)	9 (8.1)			
Agree	113 (40.5)	56 (40.0)	82 (36.3)	39 (35.1)			
Strongly agree	136 (48.7)	67 (47.9)	119 (52.7)	55 (49.5)			
I feel supported by management to break up prolonged sitting and limit the total amount of time young children spend sitting each day	*n* = 279	*n* = 135	*n* = 225	*n* = 108	−0.5 (−1.2, 0.3)	0.6 (0.3, 1.3)	0.07
Mean (95% CI)	6.1 (5.9–6.2)	6.2 (6.0–6.4)	6.1 (5.9–6.2)	6.0 (5.8–6.2)			
Strongly disagree	0 (0.0)	1 (0.7)	1 (0.4)	1 (0.9)			
Disagree	4 (1.4)	0 (0.0)	2 (0.9)	1 (0.9)			
Somewhat disagree	1 (0.4)	2 (1.5)	5 (2.2)	0 (0.0)			
Neither agree nor disagree	16 (5.7)	6 (4.4)	12 (5.3)	12 (11.1)			
Somewhat agree	31 (11.1)	12 (8.9)	20 (8.9)	9 (8.3)			
Agree	127 (45.5)	54 (40.0)	99 (44.0)	46 (42.6)			
Strongly agree	100 (35.8)	60 (44.4)	86 (38.2)	39 (36.1)			
Other educators in this service support me in breaking up prolonged sitting and limiting the total amount of time young children spend sitting each day	*n* = 278	*n* = 135	*n* = 227	*n* = 108	−0.6 (−1.3, 0.1)	0.6 (0.3, 1.1)	0.01
Mean (95% CI)	6.0 (5.9–6.1)	6.0 (5.8–6.2)	6.0 (5.8–6.1)	5.8 (5.5–6.0)			
Strongly disagree	0 (0.0)	1 (0.7)	1 (0.4)	1 (0.9)			
Disagree	4 (1.4)	0 (0.0)	1 (0.4)	1 (0.9)			
Somewhat disagree	2 (0.7)	4 (3.0)	6 (2.6)	0 (0.0)			
Neither agree nor disagree	15 (5.4)	5 (3.7)	13 (5.7)	15 (13.9)			
Somewhat agree	38 (13.7)	15 (11.1)	26 (11.5)	15 (13.9)			
Agree	131 (47.1)	65 (48.1)	107 (47.1)	47 (43.5)			
Strongly agree	88 (31.7)	45 (33.3)	73 (32.2)	29 (26.9)			

*Note*: Estimates from generalized linear mixed effects models adjusted for educator age and education.

Abbreviation: CI, confidence interval; ICC, intraclass correlation at service level.

^a^ Of educators who reported providing any screen time.

#### Policy recommendations

At baseline and post‐intervention, in both groups, nearly all educators (>96%) reported meeting the policy recommendation of young children not being seated for more than 60 min at a time (Table 2); due to the limited variation, this measure was unable to be analysed further. At baseline, 73.9% of educators in the intervention group reported providing no daily screen time compared with 59.9% of educators in the wait‐listed comparison group. However, there were no significant effects of the intervention on changes in daily screen time provided to children or the amount of time children were expected to remain seated (group‐by‐time *p*‐values >0.05; Table 2).

#### Educator practices

For the three sedentary behaviour‐related practices, the majority of educators in both groups reported health promoting (or good) practices at both time points. At baseline, 56.1% of the wait‐listed comparison and 64.8% of the intervention group reported often to always avoiding sitting while supervising outside play (Table 2). Never increasing screen time as a reward for good behaviour or taking it away for bad behaviour was reported by 83.1% of the wait‐listed comparison and 90.2% of the intervention group at baseline. At baseline, of the educators who reported providing children with screen time, 75.6% of the wait‐listed comparison and 65.2% of the intervention group reported that when screen time is offered, they often to always give children the opportunity to do an alternative activity. There were no significant effects of the intervention on changes in educator sedentary behaviour‐related practices (group‐by‐time *p*‐values >0.05; Table 2).

#### Psychosocial influences

For the three psychosocial constructs related to children's sedentary behaviour, educators in both groups reported high levels of self‐efficacy, motivation and social support at both time points. Self‐efficacy and motivation to break up prolonged sitting and to ensure children don't have sedentary screen time while in care were high at baseline in both wait‐listed comparison and intervention groups (all items and groups >90% somewhat agree to strongly agree) (Table 2). As well, social support from management and other educators to break up prolonged sitting was high for educators in both groups (both items >90% somewhat agree to strongly agree). There were no significant effects of the intervention on sedentary behaviour‐related psychosocial influences (group‐by‐time *p*‐values >0.05; Table 2).

## DISCUSSION

Minimizing young children's sitting and sedentary screen time is important for preventing child obesity and improving health and development across childhood.^4^, ^5^, ^6^, ^46^ The aim of the Play Active policy‐based intervention was to improve ECEC educator's physical activity and sedentary behaviour‐related practices. In the present study, educators in both intervention and control services reported high scores for sedentary behaviour policy recommendations, educator practices and psychosocial influences related to young children's sedentary behaviour at baseline, and thus did not significantly change in response to the Play Active policy intervention.

Almost all educators reported children did not sit for more than 60 min at a time, and around 60% did not provide children with any screen time, while less than 6% provided 15 min or more of screen time per day. The limited use of screens in ECEC is consistent with our previous cross‐sectional research of almost 1600 two–five‐year‐olds attending 104 ECEC services in Perth, Western Australia, showing less than 15% of educators allowed children more than 30 min per day of screen time.^47^

While the Play Active policy recommends ‘sedentary screen time for purposes other than learning should not be allowed’, it is possible the recommendation ‘children are not seated for more than 60 min at a time’ is too lenient and a lower limit for periods of prolonged sitting is required. This is supported by a study of 127 preschooler's device‐measured sedentary bouts in family child care homes showing children mostly do short sedentary bouts of less than 5 min with few lasting more than 10 min.^15^ Using existing wearable sensor data, future research could develop population‐referenced percentile values for children's sedentary behaviour while attending ECEC.^48^ Such age‐ and sex‐specific percentile reference values could be used to assign norm‐referenced ratings for sedentary behaviour in children attending ECEC and study relationships with young child obesity, health and development.

Most educators reported having good practices and high levels of self‐efficacy, motivation, and support in relation to managing children's sedentary behaviour at ECEC. Similar findings were observed in the evaluation of the implementation of the BC, Canada Active Play Standards whereby many physical activity and sedentary behaviour practices were reported to be high at baseline, leaving little room for improvement.^23^ While in this Canadian study and the current Australian study the majority of ECEC educators had health promoting sedentary practices, it is vital they continue to adhere to best‐practice guidelines for children's sedentary behaviour in ECEC. It is also important to highlight that sedentary practices in ECEC can vary significantly both within and between countries in response to what ECEC‐specific sedentary behaviour guidelines, policies, standards, regulations and licensing are in place and whether they are voluntary or mandatory.^17^ Accordingly, further research is needed to confirm these findings using large national movement behaviour policy intervention studies in ECEC.

While the focus of the Play Active policy was physical activity, it was developed to align with the Australian 24‐Hr Movement Guidelines for the Early Years,^49^ and thus includes recommendations and practices focused on children's sedentary behaviour in ECEC. Five of the nine age‐specific recommendations and three of the 25 practices are related to children's sedentary behaviour.^16^ Future iterations of the Play Active policy may wish to focus on additional sedentary behaviour‐related practices related to sedentary leisure‐based screen time. Furthermore, future ECEC policy development and intervention research should provide guidance on the different domains of sedentary time (i.e., reading a book vs. watching educational programs) and the impact on child obesity and other health and development outcomes. It is also important that such policies align with national and international guidelines and consider movement behaviours together rather than in isolation.

Furthermore, policy intervention research in the ECEC sector should take into account existing legislation and whether ECEC services are required to have and implement movement behaviour policies. For example, in Australia the National Childcare Regulations^50^ require services to have a separate sleep, healthy eating and sun protection policy but not a physical activity and sedentary behaviour policy. In addition, the Australian Children's Education and Care Quality Authority National Quality Framework for Early Childhood Education and Care states that ‘Each child's health and physical activity is supported and promoted’.^51^ Yet, these national standards do not provide specific information on how much physical activity (or maximum sedentary behaviour including screen time) children should have while at ECEC and offer limited resources and professional development to support educators to meet the standards. In contrast, in BC Canada where new Active Play Standards were mandated in 2017, there has been a significant increase in services with policies related to children's sedentary behaviour, and some improvement in educator sedentary behaviour practices.^23^ Longer term follow‐up measuring educator‐ and child‐level outcomes as well as providing continued implementation supports will be needed to determine the longer‐term impact of the Canadian Active Play Standards and Australian Play Active policy.

### Study strengths and limitations

Strengths of this study include an evidence‐informed ECEC‐specific movement behaviour policy intervention with a focus on children's sedentary behaviour, the large sample size and the use of educator‐based outcome measures associated with lower levels of sedentary time in children (i.e., educators psychosocial influences and practices related to children's sedentary behaviour at ECEC). This is important as the adoption and implementation of new policy is complex^18^ and can take considerable time before it has the desired impact (i.e., a reduction in children's sedentary time and/or increase in physical activity). The study was limited by the use of educator‐reported survey items which are subject to social desirability bias; however, the bias was likely present at both pre‐ and post‐intervention and thus may not have changed the intervention effect. Future studies should also consider the inclusion of device‐based measures of children's sedentary time in ECEC. Moreover, while the Play Active policy intervention included infants, there were insufficient data to examine the impact of the intervention on educator adherence to infant‐related sedentary behaviour policy recommendations and educators' practices and psychosocial influences specific to infant sedentary behaviour. Finally, the findings may not be generalizable to other countries where the ECEC sector's organizational, operational and political context is different.

### Conclusion

Adherence to sedentary behaviour policy recommendations, educator's practices and psychosocial influences related to managing young children's sedentary behaviour while attending ECEC were high at baseline and did not significantly change in response to the Play Active policy intervention. Given the value of establishing healthy behaviours early in life, it is important that children's sitting and screen time at ECEC does not increase to unhealthy levels. Implementing clear evidence informed policy, standards and legislation to minimize children's sedentary behaviours in ECEC may be an effective prevention strategy.

## AUTHOR CONTRIBUTIONS

**Hayley Christian:** Conceptualization; methodology; investigation; writing—original draft; writing—review and editing; supervision; project administration; funding acquisition. **Andrea Nathan:** Conceptualization; methodology; investigation; writing—original draft; writing—review and editing; supervision; project administration. **Emma Adams:** Methodology; formal analysis; investigation; data curation; writing—original draft; writing—review and editing; project administration. **Stewart G. Trost:** Conceptualization; methodology; writing—review and editing; funding acquisition. **Jasper Schipperijn:** Conceptualization; methodology; writing—review and editing; funding acquisition. All authors read and approved the final manuscript.

## FUNDING INFORMATION

This work is supported by the National Health and Medical Research Council (NHMRC) partnership project grant (#APP1152086) and partially through the Australian Research Council's Centre of Excellence for Children and Families over the Life Course (#CE200100025). Hayley Christian is supported by a National Heart Foundation Future Leader Fellowship (#102549) and The Kids Research Institute Australia—Ascend Senior Research Fellowship. Andrea Nathan is supported by the Australian Research Council Centre of Excellence for Children and Families over the Life Course (#CE200100025). Emma Adams is supported by an Australian Government Research Training Program Fees Offset and Stipend, a UWA and Graduate Women (WA) Research Scholarship, and a Stan and Jean Perron Top Up Scholarship. The funding bodies had no role in the design of the study and collection, analysis, and interpretation of data, and in writing the manuscript.

## CONFLICT OF INTEREST STATEMENT

No conflict of interest was declared.
